# Endovascular treatment outcomes in patients with residual cerebral aneurysmatic filling after open surgery

**DOI:** 10.1007/s10143-025-04073-0

**Published:** 2026-01-23

**Authors:** Idris Gurpinar, Onur Ozbakir, Berkay Ayhan, Yigit Can Senol, Ergun Daglioglu

**Affiliations:** 1 Neurosurgery, Koc University Hospital, Istanbul, Turkey; 2 Neurosurgery, Bilkent City Hospital, Ankara, Turkey; 3 Neurosurgery, Ankara Training and Research Hospital, Ankara, Turkey; 4 Department of Neurological Surgery, University of California San Francisco, San Francisco, California USA

**Keywords:** Residual aneurysm, Recurrent aneurysm, Clipped aneurysm, Endovascular treatment

## Abstract

Residual or recurrent aneurysmatic filling may be detected during follow-up after open aneurysm surgery and can necessitate retreatment. In this study, we descriptively report the safety and radiological/clinical outcomes of endovascular treatment (EVT) in patients who previously underwent microsurgical clipping (MSC) or surgical wrapping. We retrospectively reviewed patients treated between 2012 and 2022 at Ankara Numune Training and Research Hospital and Ankara City Hospital. Demographics, timing of initial surgery and EVT, aneurysm location, and EVT modality (flow diverter, stent-assisted coiling, primary coiling) were recorded. Clinical outcomes were assessed using the modified Rankin Scale (mRS). Radiological outcomes were assessed using the O’Kelly–Marotta (OKM) scale for flow diversion and the Modified Raymond–Roy Classification (MRRC) for coiling techniques. Procedure-related complications, mortality, and permanent morbidity were evaluated. A total of 70 aneurysms in 68 patients were treated. Complete occlusion (OKM-D or MRRC-1) was achieved in 64% of aneurysms at follow-up; when near-complete occlusion (OKM-C or MRRC-2) was included, the overall angiographic success rate was 92%. Permanent morbidity was 4.4% and mortality was 1.4%. Functional outcomes improved over follow-up, and clinical results were generally favorable across EVT techniques. EVT appears to be a feasible and effective retreatment option in selected patients with residual or recurrent aneurysmatic filling after prior open aneurysm surgery. Given the retrospective design and limited subgroup sizes, the present findings should be interpreted as descriptive. Larger prospective studies with longer follow-up are warranted to further define optimal retreatment strategies.

## Introduction

Detection of residual aneurysmatic filling on postoperative angiographic control may remain challenging for neurosurgeons, even when meticulous microsurgical techniques are employed. Despite the use of advanced intraoperative adjuncts such as indocyanine green angiography, micro-Doppler assessment, and refined microsurgical maneuvers, residual aneurysmatic filling may occasionally persist. Nevertheless, technical case reports have documented clip slippage during the postoperative period despite initially successful microsurgical clipping. Another challenge in these cases is the increased risk associated with repeat surgery due to postoperative changes at the surgical site. Most of these cases are usually detected months or even several years after the surgery, which makes the second intervention more risky due to the scar tissues and the adjunct materials such as fibrin glue and hemostatic agents used during the initial operation. Previous studies have suggested that, in selected cases, endovascular approaches may be associated with a lower procedural risk compared to repeat microsurgical intervention. Accordingly, endovascular aneurysm treatment has become an important complementary option in the management of selected cases in recent years.

The study aims to descriptively report the radiological and clinical outcomes of endovascular treatment and to document procedure-related complications in this specific patient population. Within the scope of the study, clinical experiences of endovascular treatment in cases of residual or recurrent filling in aneurysms previously treated with surgical clipping or due to technical challenges, only wrapping, will be shared. The aim of this study is to report the outcomes of endovascular treatment in patients with residual or recurrent aneurysms following prior microsurgical intervention. Moreover comparison between endovascular treatment techniques were also evaluated. The obtained results may shed light on clinical practices in the field of cerebral aneurysm treatment and guide future research endeavors.

**Fig. 1:**
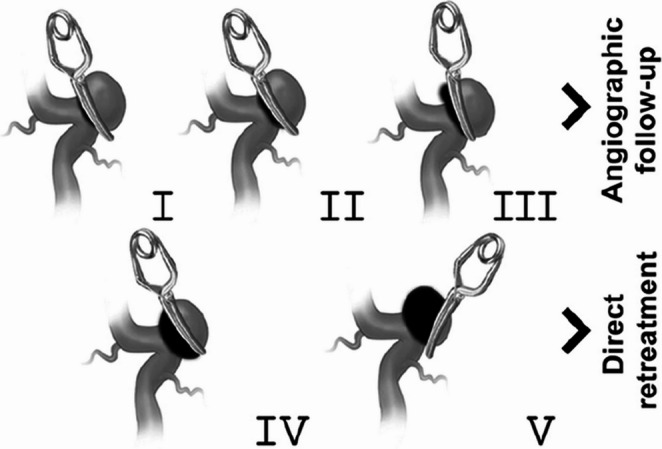
Sindou aneurysm clipping classification

This study focuses specifically on residual or recurrent aneurysms following microsurgical clipping, which represent a distinct entity with different risk dynamics compared to incidentally detected untreated aneurysms. Although the Sindou classification was originally developed to describe the immediate postoperative status after microsurgical clipping [1], its morphological criteria also provide clinically relevant information during long-term follow-up (Fig. 1). Delayed regrowth that resembles a Sindou grade 4 or 5 remnant carries similar implications for persistence and the need for retreatment. Therefore, using the same grading framework allowed us to apply a standardized and reproducible method when characterizing both immediate postoperative remnants and delayed recurrent filling in our cohort.

Even after microsurgical clipping, residual or recurrent aneurysms may persist and continue to pose a risk of delayed hemorrhage. Management of these lesions is challenging, as repeat craniotomy is often associated with increased technical difficulty due to adhesions, vascular distortion, and clip-related scarring. For these reasons, endovascular treatment (EVT) has emerged as an important alternative for selected patients requiring retreatment after microsurgical clipping or wrapping. This study aims to evaluate the radiological and clinical outcomes of EVT in this specific population and to compare different endovascular techniques used in the management of these complex cases.

## Materials and methods

Between 2012 and 2022, clinical and radiological data of patients who applied to the Ankara Numune Training and Research Hospital and, following the closure of this hospital, were transferred to the Ankara City Hospital Neurosurgery Clinic, were retrospectively examined after the approval of Ankara City Hospital Ethics Committee with decision number E1-22–3052 dated 14.12.2022. All procedures performed in studies involving human participants were in accordance with the ethical standards of the institutional and/or national research committee and with the 1964 Helsinki Declaration and its later amendments or comparable ethical standards.

### Patients and data collection

The study evaluated patient demographics, including age and gender, as well as the dates of MSC and EVT. For EVT, the specific method used (flow diverter, stent-coil, primary coil) and the anatomical localization of the relevant aneurysm were recorded. In our center, the selection of EVT modality followed a predefined practical algorithm based on aneurysm morphology and parent-artery anatomy. Primary coiling was preferred for small, narrow-necked aneurysms in which stable coil packing could be safely achieved without adjunctive devices. In wide-necked aneurysms where primary coiling was considered unsafe due to the risk of coil prolapse, stent-assisted coiling or flow diversion was selected [2]. Flow diverters were primarily used for sidewall aneurysms arising from parent arteries without major branching vessels, allowing safe parent-artery reconstruction [3]. In contrast, for bifurcation aneurysms where covering a branching vessel with a flow diverter may increase thromboembolic risk, stent-assisted coiling was favored to preserve branch patency [4]. This algorithm was consistently applied by the same neurovascular team, although final treatment choice was individualized based on surgical history, neck configuration, and antiplatelet suitability. We acknowledge that, despite these predefined principles, the retrospective design inherently limits full standardization. Additionally, the sizes of recurrent or residual aneurysms and the duration of follow-up were assessed. Data collection encompassed a comprehensive review of these parameters to provide a thorough analysis of the patient cohort. In all patients, the treatment decision was made by the same team consisting of neurosurgeons and interventional radiologists.

Preoperative and postoperative clinical information of patients was assessed and classified according to the Modified Rankin Scale (mRS). Complications arising from the procedures were examined. Perioperative and postoperative radiological imaging were evaluated, and patients were grouped according to the O’Kelly-Marotta Classification(OKM) and Modified Raymond-Roy Classification(MRRC). Postoperative follow-ups of patients were assessed at the 1 st, 6th, and 12th months based on the mRS. In the evaluation of radiological results, OKM was used for patients undergoing flow diverter (FD) treatment, while MRRC was employed for patients treated with primary coil (PC) or stent-coil (SC) therapy. At the 12-month follow-up, treatment was considered successful if the filling grade was C or D according to the OKM classification, while Class I and II were considered successful treatment according to the MRRC classification.

## Inclusion and exclusion criteria

A total of 1442 patients who had previously undergone aneurysm treatment with microsurgical clipping (MSC) or “wrapping” were initially examined. In follow-up Digital Subtraction Angiography (DSA) imaging, cases classified as grade 1, 2, and 3 according to the Sindou Classification were excluded from the study. In this study, the Sindou classification was used as a standardized angiographic framework to describe the extent of aneurysmatic filling at the time of follow-up imaging and to guide retreatment eligibility, rather than to assess the technical success or durability of the initial microsurgical clipping. However, re-treatment was decided in only 5 patients with grade 3 due to the presence of an aneurysmal bleb. Among them, 76 patients who were identified with residual or recurrent aneurysms during follow-ups and subsequently received EVT were included in the study. 8 patients in this group who either lacked postoperative follow-ups or did not have a DSA at the 12-month were excluded from the study. Therefore, the clinical and radiological outcomes of a total of 68 patients were evaluated in this study. Accordingly, the proportion of patients requiring endovascular retreatment corresponds to a small, highly selected subgroup (76 of 1442 patients, 5.3%) and should not be interpreted as a recurrence rate of microsurgical clipping. Rather, this cohort represents cases in whom residual or recurrent aneurysmatic filling was identified during follow-up and deemed to warrant additional treatment.

Patients who had previously undergone surgical wrapping were included in the cohort, acknowledging that wrapping represents a non-curative, protective surgical strategy that is fundamentally distinct from microsurgical clipping. These cases were included because they similarly present with residual aneurysmatic filling requiring retreatment. However, due to the limited number of wrapping cases (*n* = 5), separate subgroup analysis was not statistically feasible.

### Statistical analysis

IBM SPSS Statistics for Windows, Version 26.0, was utilized for the evaluation of data obtained in the research. The Shapiro-Wilk normality test was applied to assess the normality of variables within specific group factors. One-way Analysis of Variance (ANOVA) or Kruskal-Wallis Analysis of Variance techniques were employed for the assessment of continuous measurements among more than two independent groups. Homogeneity tests for categorical variables were conducted using the chi-square test and/or Fisher’s exact test. For examining time-dependent changes among groups, Repeated Measures ANOVA models were used. Greenhouse-Geisser test statistics and corresponding p-values were utilized based on Mauchly’s sphericity test results. Results obtained from these models, including time-dependent changes, inter-group differences, and group-time interaction, were summarized using tables and graphs. Descriptive statistics for continuous variables were presented as mean ± standard deviation and median (25% − 75%), while frequency distributions and percentages were provided for categorical variables. A significance level of *p* < 0.05 was used for evaluating the results of the study. Clinical outcomes were evaluated using the modified Rankin Scale (mRS). Because mRS represents an ordinal variable, descriptive data were expressed as median and interquartile range (IQR) rather than mean ± standard deviation. Between-group comparisons of mRS scores were performed using the Kruskal–Wallis test. In addition, functional outcome was dichotomized as favorable (mRS 0–2) versus unfavorable (mRS 3–6), and categorical comparisons were conducted using the chi-square or Fisher’s exact test, as appropriate. Although 1-month mRS values were reported to document early postoperative neurological evolution, the 12-month mRS score was used as the sole endpoint for final outcome analysis, consistent with standard reporting in neurovascular studies.

## Results

### Demographic, Clinical, and radiological results

A total of 68 patients meeting the criteria were included in our study. Among these 68 patients, a total of 70 aneurysms were treated using endovascular techniques. Five of these aneurysms were initially treated with the “wrapping” surgical technique due to technical or clinical challenges during the first operation. Out of the 68 patients, 44 were female (64.7%), and 24 were male (35.3%). Regarding the classification based on aneurysm localization, there were 21 aneurysms (30%) in the ACA group, 13 aneurysms (18.57%) in the ICA group, 34 aneurysms (48.58%) in the MCA group, and 2 aneurysms (2.85%) in the PCOM group. Given the limited sample sizes within individual territories—particularly PCOM—location-based comparisons should be interpreted as descriptive rather than definitive. The youngest treated patient was 31 years old, and the oldest was 78 years old (average age: 52.11 ± 10.96). The average age of patients was also similar when evaluated according to aneurysm groups (Table 1).

**Table 1:** Overview of patients (*Chi-square test shows the p-value, while all others show p-values of fisher’s exact test. **Bif: Bifurcation)

Parameters	Stent-Coil	FD	Primer-Coil	*p*-value
Age	50.72 ± 12.06 49(41–60)	52,25 ± 9,95 51(44,5–59)	55,11 ± 10,2 51(49–58)	0.938*
Aneurysm Weight	5.48 ± 1.99 5,5(4–6,4)^a^	4,32 ± 3,16 3(2,2–5,5)^b^	4,62 ± 1,76 4(3–6)^ab^	*0.006*
Aneurysm Height	5.01 ± 2.81 5(3–6)	3,92 ± 2,46 2,9(2,5 − 4,7)	4,26 ± 1,56 3,5(3–5)	0.120
Dome-Neck Ratio	1.43 ± 0.88 1,2(0,8 − 1,9)	1,45 ± 0,72 1,4(1,0–1,6)	1,95 ± 1,37 1,5(1,0–2,1)	0.482
		ACA	ICA	MCA
Gender	Male	9(%42,86)	4(%30,77)	11(%32,35)
	Female	12(%57,14)	9(%69,23)	23(%67,65)
SAH	No	5(23,81)	5(38,46)	13(38,24)
	Yes	16(76,19)	8(61,54)	21(61,76)
Endovaskular Embolization	Stent-Coil	13(%61,9)	0(0)	16(47,06)
	FD	3(14,29)	12(92,31)	16(47,06)
	Primer-Coil	5(23,81)	1(7,69)	2(5,88)

(*) p-value refers to the ANOVA test, while the others denote the p-values of the Kruskal-Wallis test. ^a, b, ab^; medians sharing the same superscript are identical, whereas those with different superscripts are statistically different. *Statistically significant at *p* < 0.05

**Fig. 2:**
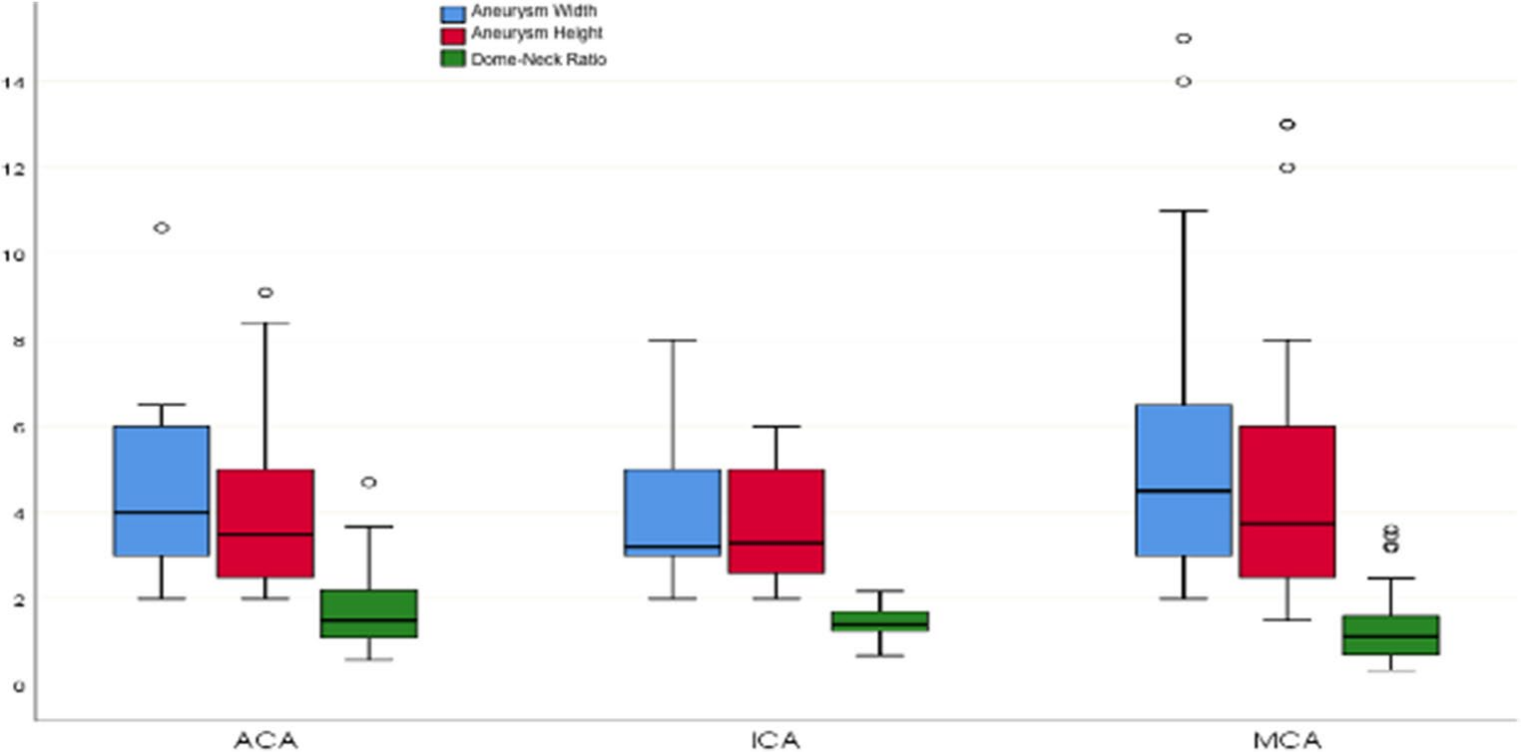
Graphical view of aneurysm sizes according to aneurysm localization

The duration between the first treatment (MSC or “wrapping”) and the second treatment (EVT) ranged from a minimum of 1 month to a maximum of 192 months (53.6 ± 39.9). In the initial surgery, 38 patients (55.88%) presented with subarachnoid hemorrhage (SAH), while 8 patients (11.76%) presented with SAH before the second surgery. Due to the unavailability of post-first-operation DSA images in the archive records for each patient, the Sindou Clipping Grade was assessed from the DSA performed during the second presentation. As a result, it was not possible to reliably distinguish true late recurrence from initially incomplete clipping that became apparent during delayed follow-up. This limitation precludes direct comparison of recurrence rates with contemporary microsurgical series and reinforces that the present study does not assess clipping durability. Accordingly, 44 patients (67.69%) were classified as Grade 5, 21 patients (32.3%) as Grade 4, and 5 patients as Grade 3. When comparing aneurysm sizes during the second presentation, the average aneurysm width was 4.24 ± 2.6 mm, and the average aneurysm height was 4.42 ± 2.53 mm. The Dome-Neck Ratio, representing the ratio of aneurysm height to neck diameter, ranged from a minimum of 0.34 to a maximum of 4.7 (1.50 ± 0.89) (Fig. 2).

### Endovascular treatment: clinical and radiological outcomes

A total of 70 aneurysms were treated in 68 patients, with 2 of them was PCOM aneurysms. Due to the small sample size, this group was not included in the statistical calculations and Table 1. Out of these aneurysms, one of them was successfully treated with FD, showing positive radiological and clinical outcomes (Preoperative OKM: A1, 12th-month OKM: D). The other was initially treated with primary coiling; however, the patient presented with subarachnoid hemorrhage (SAH) six years after endovascular treatment, leading to re-coiling. Vazospasm developed during the procedure, and despite the second endovascular treatment, significant clinical improvement could not be achieved at 6 months (mRS 1 st month: 4/6th month: 3/12th month: 3), but radiological improvement was observed (Postop MRRC: 2–12th month MRRC: 1). Of the remaining 68 aneurysms, 29 were treated with stent-coil, 31 with flow diverter (FD), and 8 with the primary coiling method.

#### Clinical outcomes

**Fig. 3:**
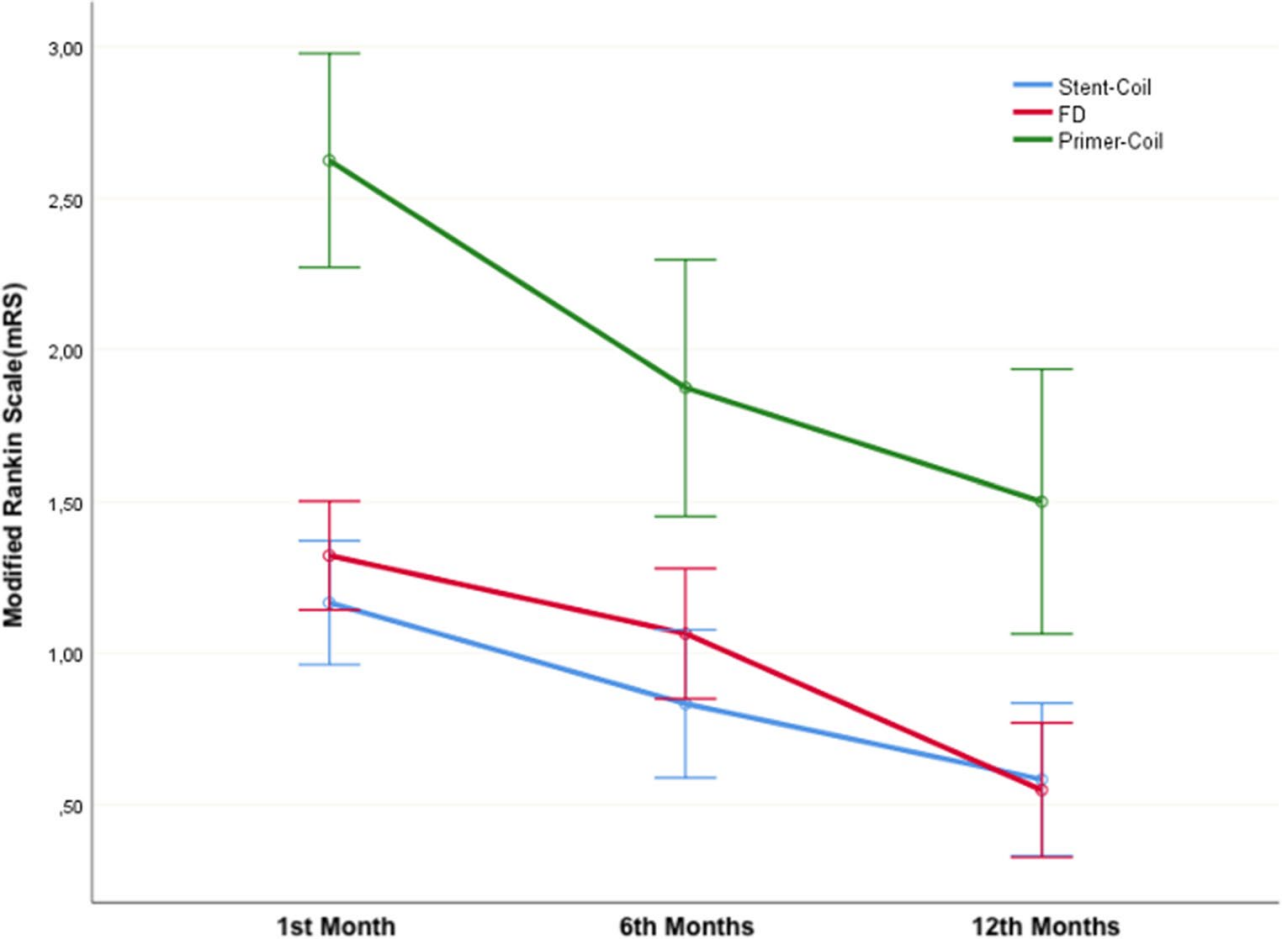
Evaluation of Modified Rankin Scale by Treatment Types

When the Modified Rankin Scale (mRS) values were examined, no statistically significant difference was found among the groups. However, independently of the groups, a statistically significant improvement in mRS scores was observed in postoperative follow-ups (*p* < 0.001). While successful clinical improvement was achieved with the treatment methods (*p* < 0.001), when the groups were compared, it was observed that they moved in parallel to each other, and statistically, there was no significant difference between the groups (p3:0.247) (Fig. 3).

**Fig. 4:**
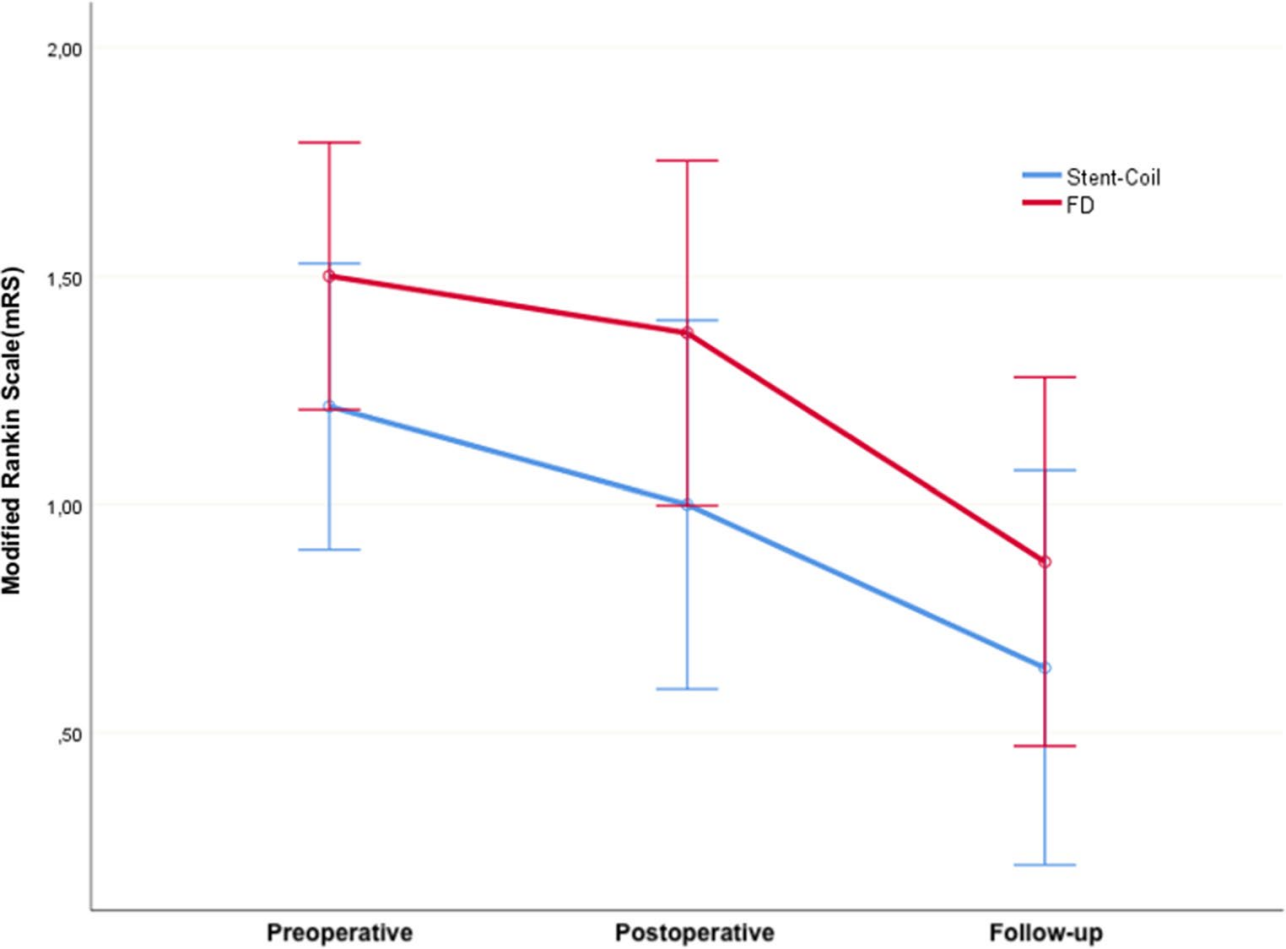
Evaluation of mRS results of two groups (stent-coil and fd) in the treatment of MCA aneurysms

The results of aneurysm treatment based on anatomical localization in the ACA, ICA, and PCOM groups could not be evaluated due to the small sample size. However, in the MCA group, 16 aneurysms were treated with FD, while 16 aneurysms were treated with stent-coil. Radiological outcomes were not evaluated due to different scales (OKM-MRRC), but clinical outcomes were assessed between these two groups. As observed in the table, there was no significant difference in treatment clinical outcomes between the groups. In the mRS graph, there was no statistically significant difference between the results (Fig. 4).

### Radiological outcomes

**Fig. 5:**
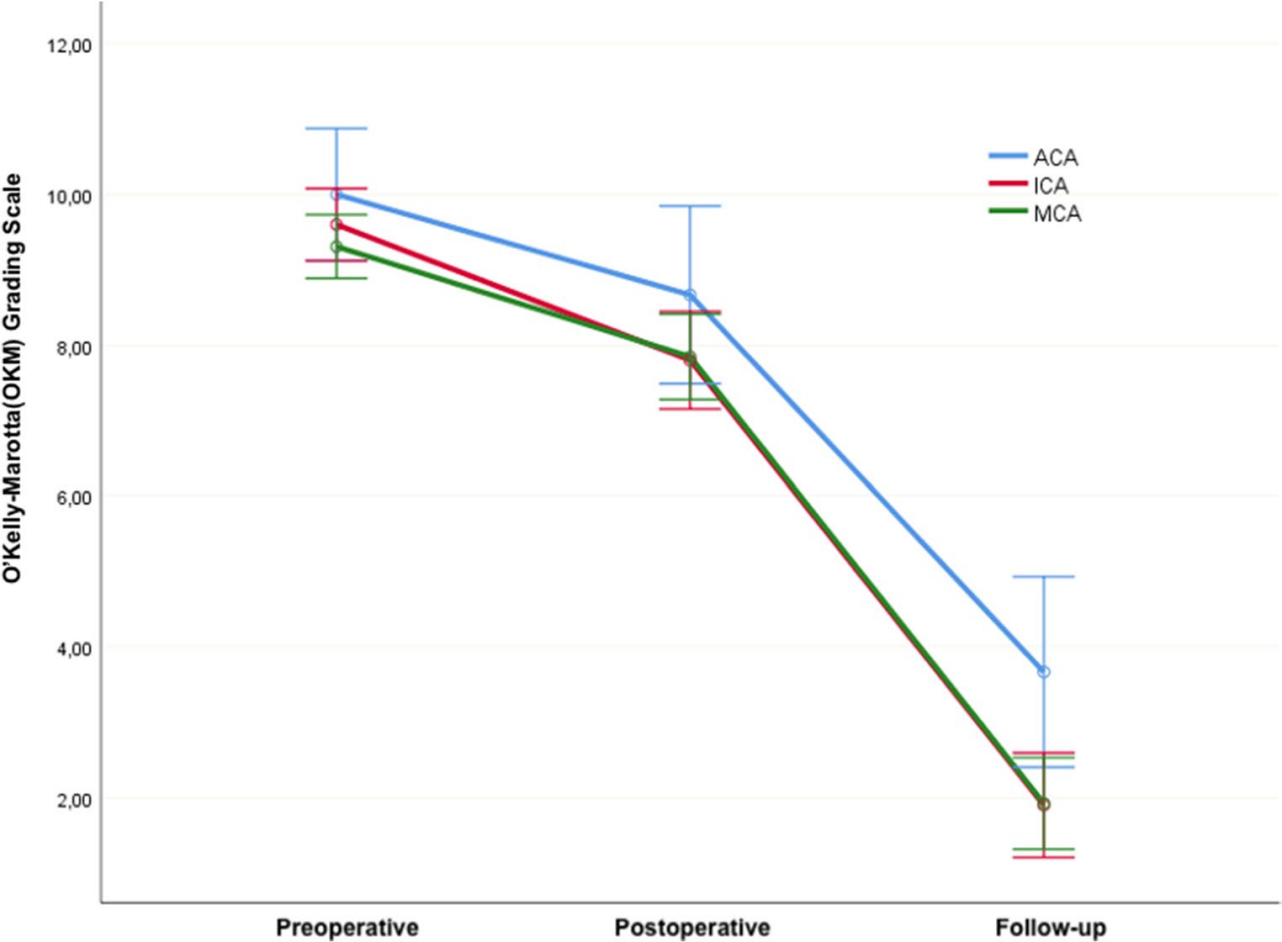
Evaluation of preoperative, postoperative, and follow-up OKM scores according to aneurysm localization

The OKM measurements for the FD group were analyzed with respect to aneurysm groups. The explanation of the two-way repeated measures ANOVA model is provided below. Comparison in patients treated with FD was analyzed using a Repeated ANOVA model, testing for differences between groups, changes over time, and group-time interaction. Accordingly, no significant difference was observed between groups (p2 = 0.485). However, a significant change over time was found in OKM measurements (p1 < 0.001). In other words, OKM improvement showed no difference concerning the anatomical localization of aneurysms treated. Furthermore, successful radiological outcomes were observed throughout follow-up, regardless of anatomical site. The group-time interaction was not significant. (p3 = 0.836) (Fig. 5).

**Fig. 6:**
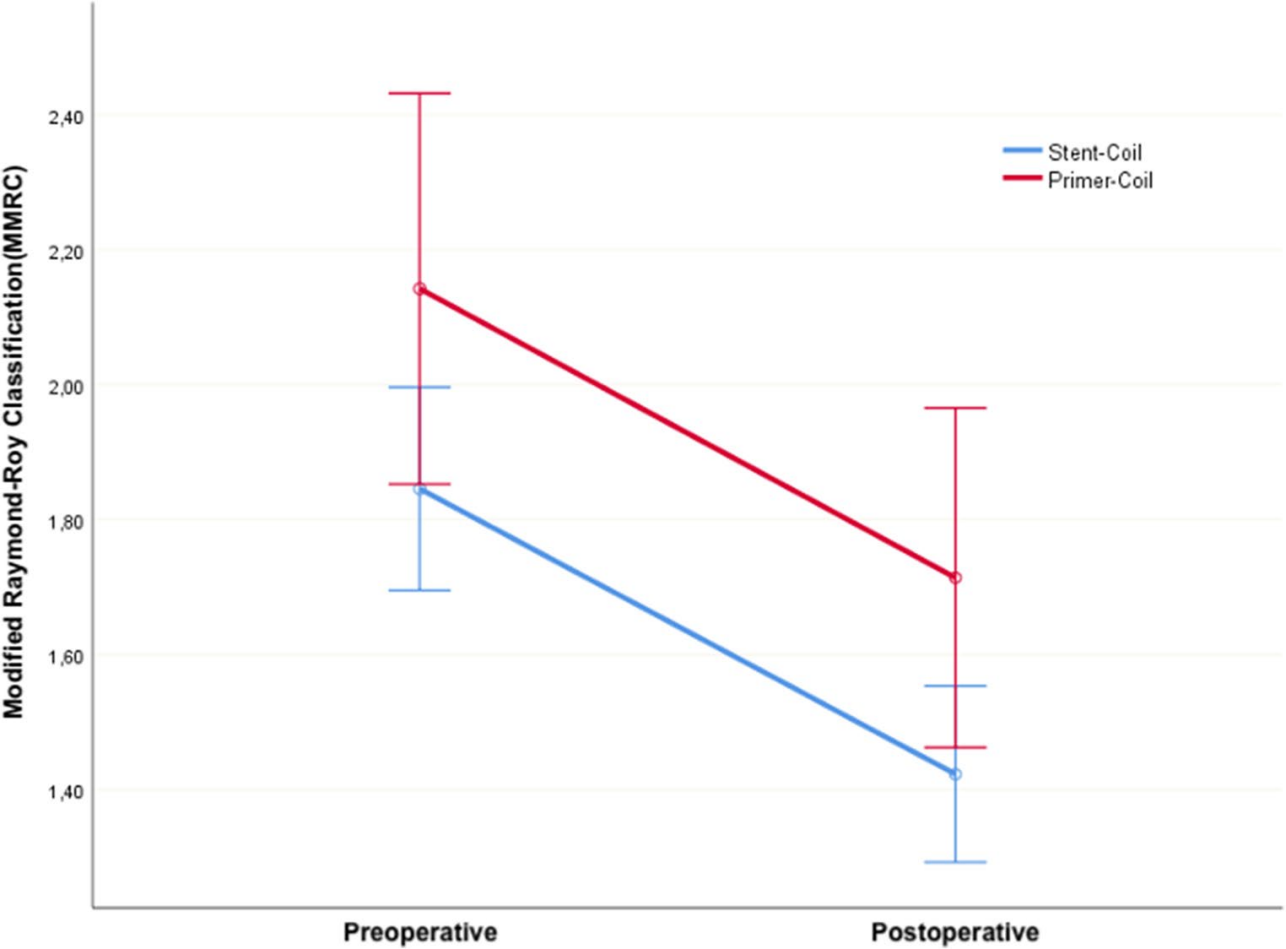
Evaluation of Postoperative and Follow-up MRRC Scores

When evaluating the MRRC measurements in the early postoperative and follow-up periods, no statistically significant difference and group-time interaction were found between the groups (p2: 0.255 and p3: 0.987, respectively). Therefore, it can be stated that the groups behaved similarly over time, and there was no difference between them. However, a significant difference was found between the early postoperative and follow-up periods (p1 < 0.001). This indicates that there was a statistically significant improvement in radiological outcomes after endovascular treatment in both groups (Fig. 6).

## Complications

Among the 68 treated patients, aneurysm rupture occurred in only one case (%1.47) after flow diverter treatment, and decompressive craniectomy was performed. The patient died during the intensive care unit follow-ups at the 3rd postoperative month due to sepsis. In two patients (%2.94) treated with a flow diverter, decompressive craniectomy was performed after endovascular embolization due to pre-existing intraparenchymal hematoma. In both cases with pre-existing intraparenchymal hematoma, the hemorrhages were located deep within eloquent brain regions and did not present a safe surgical corridor for evacuation. The primary clinical issue at presentation was malignant cerebral edema and refractory intracranial hypertension; therefore, emergent decompressive craniectomy was performed as a life-saving intervention. Hematoma removal was not attempted because the radiological characteristics (deep location, diffuse margins, and infiltration of functionally critical parenchyma) indicated that surgical evacuation would pose a high risk of additional neurological injury without clear clinical benefit. In 1 patient (1.47%) who underwent MSC surgery for a ruptured right MCA aneurysm but where only wrapping could be applied due to technical problems, left hemiplegia developed following primary coiling due to a thromboembolic event. At the patient’s 1-year follow-up, mild paresis persisted after physical therapy and rehabilitation. Two patients (2.94%) treated with primary coiling underwent repeat endovascular treatment due to recanalization, one at the 4th year and the other at the 6th year. One patient (1.47%) treated with stent-coiling was re-embolized with flow diverter due to residual filling detected on postoperative 6-month control DSA imaging.

In two patients, aneurysm recurrences were observed in two different anatomical locations that had previously been treated with microsurgical clipping. The aneurysm fillings in these patients were successfully treated endovascularly in separate sessions. Therefore, a total of 70 aneurysms were treated in 68 patients in our study. During the perioperative period, coil prolapse occurred in 2 patients (%2.94). In both cases, a new stent was placed in the parent artery. No complications related to coil prolapse were observed in either patient. Intraoperative aneurysm rupture did not occur in any patient. No patient experienced contrast-related allergic reactions or kidney failure.

Complete occlusion (OKM-D, MRRC-1) was achieved in 45 aneurysms (64%) during the initial session, while near-complete occlusion (OKM-C, MRRC-2) was also included in this group, resulting in a total of 65 aneurysms and a 92% occlusion rate at the final follow-up.

**Fig. 7:**
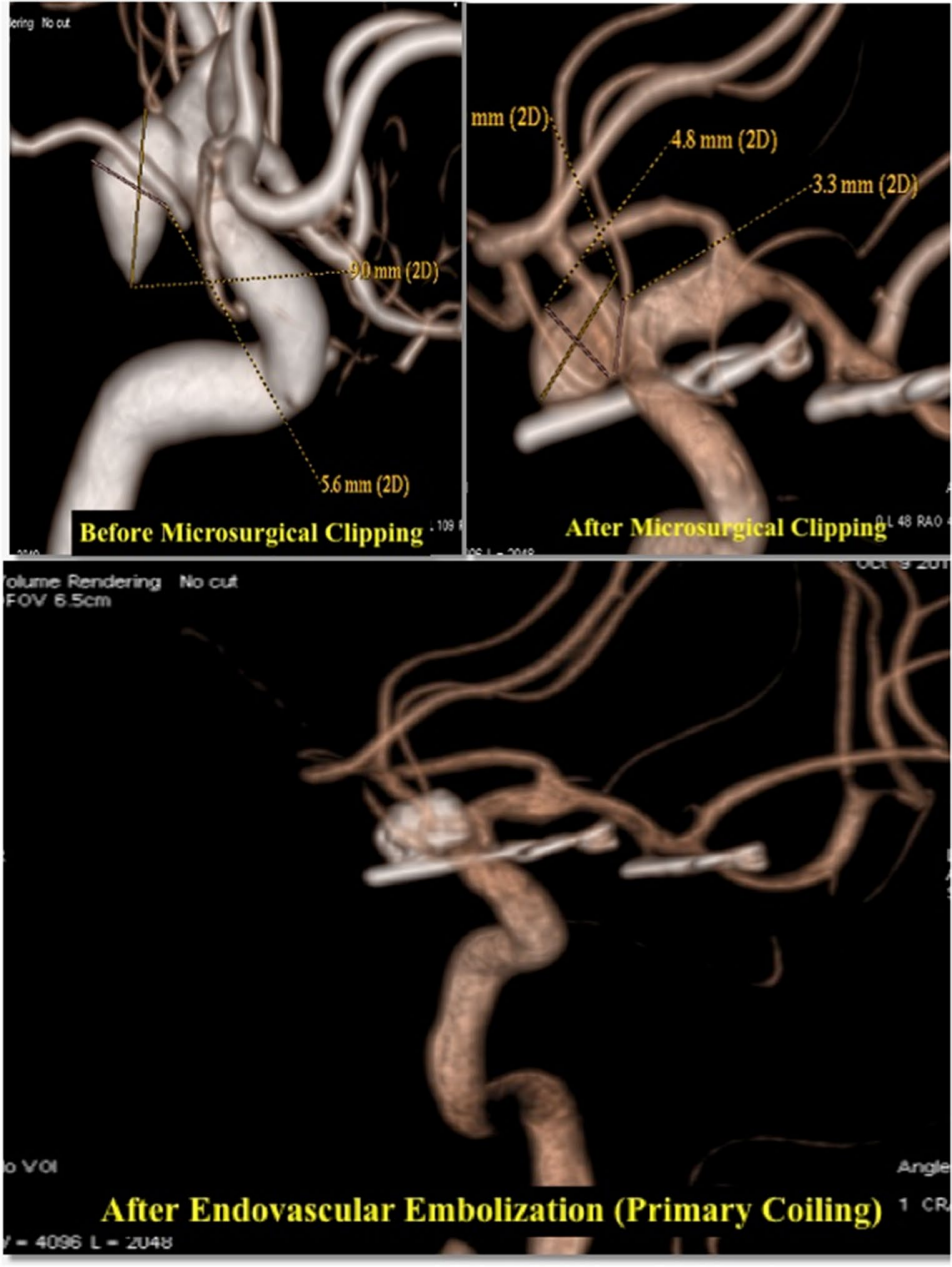
Residual PCOM Aneurysm Treated with Primary Coiling

**Fig. 8:**
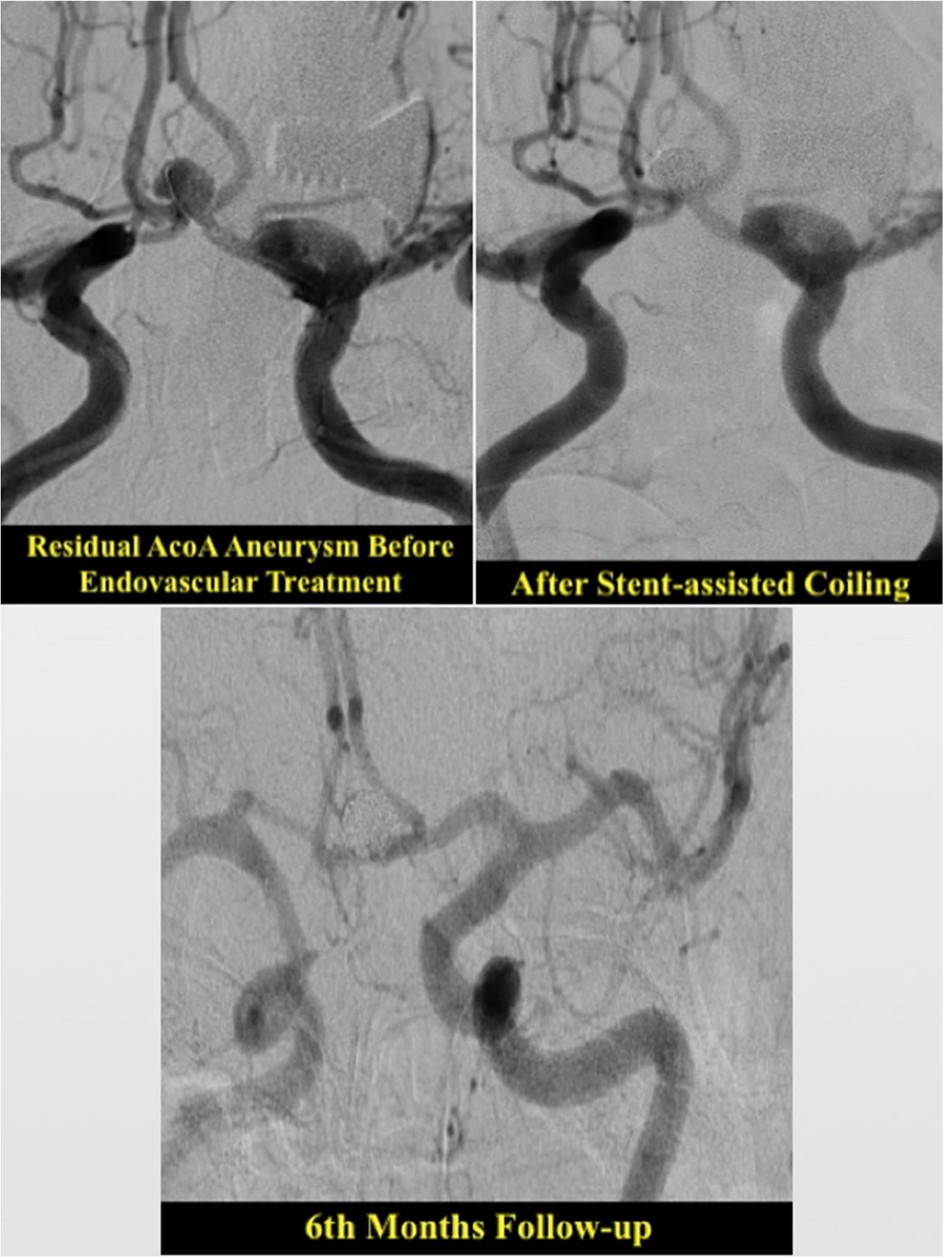
Residual AcoA Aneurysm Treated with Stent-assisted Coiling

An illustrative example of a residual PCOM aneurysm treated with primary coiling is provided in Fig. 7. Figure 8 demonstrates the endovascular stent-assisted coiling of a residual AcoA aneurysm.

## Discussion

Residual or recurrent aneurysms after microsurgical clipping may arise from technical or anatomical challenges during surgery, clip displacement related to insufficient occlusion force, regrowth from an inadequately clipped neck, or, rarely, true recurrence after apparent complete occlusion [5–7]. Although the Sindou classification was originally developed to describe the immediate postoperative status after microsurgical clipping, its morphological categories also provide valuable prognostic information during long-term follow-up. Delayed regrowth that morphologically corresponds to a Sindou grade 4 or 5 remnant exhibits a similar risk pattern for persistence or rebleeding and is therefore managed in our practice as clinically equivalent to immediate postoperative remnants. For this reason, applying the Sindou grading system to both immediate and delayed presentations allowed us to maintain a standardized and reproducible method for defining the extent of aneurysm filling that warranted retreatment. The incidence of residual aneurysms after microsurgical clipping (MSC) varies between 4% and 19%, and the incidence of subarachnoid hemorrhage (SAH) due to residue has been reported to reach up to 0.8% in some studies [7–10]. A case series evaluating only residual aneurysms after endovascular treatment revealed that previously ruptured aneurysms are at a higher risk of bleeding compared to aneurysms that have never bled [11]. Even if the size of the aneurysm residue is small, the risk of bleeding persists over the years and can lead to fatal outcomes [6, 12]. Therefore, in our clinical practice, endovascular treatment has been considered for patients with residual or recurrent aneurysms, taking into account individual anatomical and clinical factors. According to the International Subarachnoid Aneurysm Trial (ISAT) published in 2002, the risk of rebleeding from a ruptured aneurysm one year after microsurgical clipping was zero in 1081 patients [13]. However, in longer-term follow-ups of ISAT patients, Campi et al. reported in 2007 that retreatment was applied to 39 out of 1012 patients (%3.8) after microsurgical clipping [14]. This retreatment rate is numerically comparable to the proportion observed in our cohort, particularly when differences in patient selection, follow-up protocols, and imaging availability are taken into account. Tsutsumi et al. reported that the cumulative rate of subarachnoid hemorrhage (SAH) recurrence after complete obliteration of an aneurysm was 2.2% at 10 years and 9% at 20 years after the original treatment [15]. Drake et al. conducted a study on 115 patients who had previously undergone MSC, reporting a mortality rate of 5.2% and a morbidity rate of 10% in patients who underwent repeat MSC treatment due to residual cerebral aneurysms. In our study, which had an average follow-up period of approximately 4 years, the mortality rate was found to be 1.47% and permanent morbidity was 4.4%. Of course, the difference may be attributed to the older date of this study and the evolution of surgical technology over the years. However, in the setting of repeat interventions, endovascular treatment may represent a feasible alternative in carefully selected patients, particularly when reoperation carries increased technical risk. The same study reported a high risk of bleeding if an aneurysm residue or recurrence is detected, emphasizing the necessity of a second treatment [6]. Importantly, the present study was not designed to evaluate the durability of microsurgical clipping but rather to descriptively report endovascular treatment outcomes in a highly selected subgroup of patients with residual or recurrent aneurysmatic filling. Therefore, the reported retreatment proportion should be interpreted as a reflection of case selection and follow-up practices rather than as evidence of an unusually high failure rate of microsurgical clipping.

Previous studies have demonstrated that the biological behavior of recurrent or residual aneurysms after microsurgical clipping varies according to arterial location and patient-specific factors. Jabbarli et al. reported in a large multicenter cohort that recurrence rates differ significantly among arterial territories, following the gradient ACA > ICA > posterior circulation > MCA, with MCA aneurysms demonstrating the lowest recurrence risk. The same study also identified aneurysm size > 12 mm and patient age > 45 years as independent predictors of recurrence [1]. These findings help contextualize the heterogeneity observed in residual or recurrent lesions after clipping and emphasize the need for individualized retreatment strategies. Repeat microsurgical clipping, although effective in selected cases, may be technically challenging due to adhesions, clip scarring, and altered vascular anatomy [15]. Consequently, EVT has increasingly been considered as an alternative treatment modality when the risks of re-craniotomy are deemed substantial.

Although EVT has become an increasingly utilized option for the management of residual or recurrent aneurysms, modern microsurgical clipping continues to demonstrate excellent durability and safety when performed in experienced, high-volume centers. Advances such as high-resolution operative microscopy, intraoperative indocyanine green videoangiography (ICG-VA), micro-Doppler assessment, and refined clip designs have substantially improved intraoperative control of aneurysm occlusion and vessel patency [16–18]. Large contemporary series of unruptured middle cerebral artery aneurysms treated with open microsurgery have reported complete or near-complete aneurysm occlusion in approximately 90–95% of cases, with very low rates of procedure-related morbidity and no surgical mortality in high-volume practices [17]. Similarly, multimodal monitoring strategies that combine ICG-VA, microvascular flowmetry, and neurophysiological monitoring have achieved aneurysm exclusion rates close to 99% with low symptomatic ischemic complications [18]. These data underline that EVT should be regarded as a complementary modality rather than a replacement for modern microsurgical practice, particularly in patients in whom reoperation is considered high risk because of scarring, clip adherence, or complex vascular anatomy [16, 17].

The first significant case series was published by Rabinstein et al. in 2002, involving 21 patients. Total closure was achieved in 81% of the 21 patients who underwent primary coil or balloon-assisted coil treatment [19]. Subsequently, treatment results related to coil embolization were evaluated by others, and it was reported that the treatment method is safe and successful. However, these studies only evaluated primary coil treatment or balloon-assisted coil treatment. In our study, stent-assisted coil and flow-diverting stent treatments were also practiced to patients. Similar to the literature, our study found statistically significant improvement in both clinical and radiological follow-up parameters in the group of patients who underwent only primary coil treatment.

The largest case series to date is a study conducted in Brazil in 2021, managed in 5 different centers, including the treatment results of 70 residual or recurrent aneurysms [20]. In this series, 27% underwent primary coil treatment, 35% underwent balloon-assisted coil treatment, 11% underwent stent-coil treatment, and 25% underwent flow-diverting treatment. When all aneurysms were evaluated, complete closure was achieved in 75.3% of the controls performed in the first year after treatment. Post-endovascular treatment aneurysmal refill was observed in 14.5% of cases. Permanent morbidity was 2.9%, and mortality was 1.4%. In our study, complete closure was achieved in 64% of the controls at 12 months. When minimal neck filling was included, nearly complete closure was achieved in 92%. Permanent morbidity was 4.4%, and mortality was 1.4%, similar to the previous publication. When similar case series are evaluated, it is observed that the rate of complete closure varies between 53% and 86%, and procedure-related morbidity and mortality range from 1% to 5.3% [21–23]. Our study is a single-center study with the largest case series to date [20] with the same number of aneurysms. When the success of treatment and overall mortality and morbidity rates are examined, similar clinical and radiological results have been obtained. In contrast to previous studies, MCA aneurysms treated with different types of endovascular treatment were evaluated within themselves, and their clinical outcomes were compared. When two different groups (SC and FD) were evaluated in terms of clinical outcomes, no statistically significant difference was observed. However, statistically successful clinical results were achieved in both groups. (Fig. 4) Unlike the previous study, in our study, endovascular treatment was performed after the wrapping technique in 5 patients. When the patient group who underwent wrapping technique is excluded from the statistical analysis, the morbidity rate decreases to 3.03% in 65 aneurysms. Patients with residual aneurysmatic filling after microsurgical clipping and those initially treated with surgical wrapping were included in the same cohort because both groups ultimately represent cases requiring retreatment. Nevertheless, surgical wrapping is a non-curative technique and constitutes a distinct clinical entity with different biological behavior and risk profiles compared with clipped aneurysms. The limited number of wrapping cases in our series (*n* = 5) precluded a meaningful statistical subgroup analysis. Notably, when patients treated with wrapping were excluded from the analysis, the permanent morbidity rate decreased from 4.4% to 3.03%, suggesting that inclusion of wrapping cases did not artificially improve outcomes and, if anything, resulted in a more conservative estimate of EVT-related morbidity. In addition, reporting only mRS outcomes and angiographic occlusion rates does not allow definitive conclusions regarding the clinical efficacy or long-term durability of EVT in preventing rupture, rebleeding, or the need for retreatment. Aneurysm-specific longitudinal endpoints such as rupture-free survival, rebleeding-free survival, and retreatment-free survival would be required to determine whether EVT truly alters the natural history in this population. However, due to the retrospective design, limited sample size, and relatively short follow-up in some cases, these outcome measures could not be reliably assessed. As such, our findings should be interpreted as descriptive observations rather than evidence of long-term clinical effectiveness.

When the treatment results in all aneurysms treated with EVT are examined, compared to our study, similar complete closure rates and lower rates of recurrence and residue were encountered in the literature. In a study conducted by Pierot et al. in 2010, they reported total aneurysm occlusion in 63%, neck residues in 22.5%, and aneurysm residues in 14.6% of cases [24]. In another study, it was revealed that 28.6% of aneurysms had recurrence at an average follow-up of 12 months, and 5.5% of these patients were found suitable for retreatment [25]. However, making a direct comparison with these studies can be misleading. This is because both the target patient population in our study is different and the fact that flow-diverting stents were not widely used in treatment at the time these studies were published.

In our study, complete closure was achieved in 27 (87%) of the 31 patients treated with a flow-diverting stent. This is similar to other case series that include remaining aneurysms after clipping and coiling, exhibiting a complete closure rate ranging from 93.3% to 100% one year after the placement of FD stents [26, 27]. In a meta-analysis of three major studies evaluating aneurysms treated with FD stents, an 85.5% complete closure rate at 1 year was reported. However, these studies did not specifically assess FD stent treatments for cases with recurrence or residue after MSC. When reviewing the literature, we identified two studies with a similar patient population to our study. The first one, conducted by Romagna et al. in 2018, treated six patients with a history of MSC and SAH with FD stents. Complete aneurysm closure was achieved in 5 (83%) patients, with no mortality or permanent morbidity observed. One patient underwent repeat endovascular treatment due to residue [28]. In a study conducted in Brazil in 2021, 18 patients with a history of MSC were treated with FD stents, achieving a 100% treatment success in follow-ups after 1 year [20]. Although the success rate in our study was even higher than the first study mentioned, it had a lower success rate than the second study. We think that this difference is due to the higher number of patients and longer follow-up period.

The group of patients previously treated with the “wrapping” technique, although the risk of rupture may decrease, the results are not curative. Therefore, these are pathologies that require re-treatment. In our study, in addition to the mentioned studies, the group of aneurysms previously treated with “wrapping” was treated by us using an endovascular approach, and successful radiological results were achieved in 5 patients. Only in one patient, hemiparesis developed after endovascular treatment, and at 1-year follow-ups, a residual paresis persisted.

Our study is the largest single-center case series evaluating the results of endovascular treatment in cases of recurrence or residue aneurysms previously treated with MSC or the “wrapping” technique. In terms of treatment technique, having the same team perform the treatment provides a form of randomization in the treatment methods. This study has several important limitations. Foremost, the absence of a control group—such as re-clipping, conservative observation, or natural history—precludes any comparative or causal inference regarding the relative efficacy or safety of EVT. As a retrospective descriptive cohort, our data should not be interpreted as evidence that EVT is superior or equivalent to microsurgical re-intervention or other management strategies. Accordingly, the present results should be interpreted as descriptive feasibility and outcome data in a selected retreatment population, rather than as a basis for recommending EVT over re-clipping or conservative management. The outcomes reported herein therefore represent observational findings rather than hypothesis-testing results, and they should be considered hypothesis-generating. Future prospective studies with matched or randomized control groups are required to determine the optimal treatment approach for residual or recurrent aneurysms following microsurgical clipping or wrapping. Another important limitation of the present study is the lack of multivariate analysis, survival analysis, or statistical adjustment for potential confounders. Owing to the retrospective design and the limited number of outcome events, advanced modeling was not feasible, which restricts our ability to account for the influence of variables such as aneurysm morphology, prior surgical treatment type, and individual comorbidities. As a result, no causal inference can be drawn from the observed associations. Prospective studies with larger sample sizes and adequate statistical power are required to allow comprehensive multivariate and time-to-event analyses. In addition, the limited sample size inevitably constrains the statistical power of subgroup analyses stratified by treatment modality and aneurysm location. Moreover, detailed morphology-based stratification (e.g., wide-neck configuration, bifurcation anatomy, or residual neck patterns) could not be performed in a standardized manner due to the retrospective design and incomplete uniform documentation of these variables across the entire study period. Therefore, we did not conduct morphology-driven risk stratification analyses, and future multicenter studies with standardized morphologic reporting are needed to address this question. Although these comparisons provide clinically meaningful descriptive information, they should be interpreted with caution and considered exploratory rather than confirmatory. The observed trends may help generate hypotheses but cannot establish statistically robust differences between subgroups. Larger multicenter cohorts are needed to allow adequately powered subgroup analyses and to validate the patterns observed in our study. However, when evaluating the patient population, the fact that all aneurysms were anterior circulation aneurysms and did not provide any experience or clinical results for posterior circulation aneurysms can be considered as limitations of this study. Additionally, the absence of early DSA images after the patients’ initial surgeries (MSC or “wrapping”) has limited investigations into the causes of residue or recurrence.

## Conclusion

The group of patients who had previously undergone MSC or wrapping treatment but presented with residual or recurrent aneurysm filling has historically been a challenging cohort for neurosurgeons specializing in neurovascular cases. Our study demonstrates that even in such a challenging patient group, satisfactory levels of safety and effectiveness in treatment were achieved both clinically and radiologically. Furthermore, our single-center study holds significance for contributing to the existing knowledge. The positive aspects of endovascular embolization treatment not only ensure successful completion of patient treatments but also alleviate the treatment burden associated with repeated microsurgical clippings. With ongoing technological advancements in endovascular embolization, outcomes may further improve. Our findings support EVT as a feasible and effective retreatment option in selected patients with residual or recurrent aneurysmatic filling after open surgery; however, comparative studies are required to define the optimal strategy versus re-clipping, observation, or other management approaches. As larger patient groups are included in future studies and longer-term results are published, the reliability of the obtained results will increase. This will contribute to determining the most effective and safe approaches for the treatment of recurrent aneurysms.

## Data Availability

No datasets were generated or analysed during the current study.
